# Promoting Child Health Equity through Health Literacy

**DOI:** 10.3390/children10060975

**Published:** 2023-05-30

**Authors:** Shuaijun Guo, Lucio Naccarella, Elisha Riggs

**Affiliations:** 1 Centre for Community Child Health, Murdoch Children’s Research Institute, Royal Children’s Hospital, Melbourne, VIC 3052, Australia; 2 Department of Pediatrics, University of Melbourne, Melbourne, VIC 3052, Australia; 3 Melbourne School of Population and Global Health, University of Melbourne, Melbourne, VIC 3053, Australia; l.naccarella@unimelb.edu.au; 4 Intergenerational Health, Murdoch Children’s Research Institute, Royal Children’s Hospital, Melbourne, VIC 3052, Australia; elisha.riggs@mcri.edu.au; 5 Department of General Practice, University of Melbourne, Melbourne, VIC 3000, Australia

Every child has the right to a fulfilling and thriving life. Often, this is not the case in the real world. Inequities in children’s health are unjust and avoidable, carrying high costs for individuals and society [1]. Addressing child health inequities is a significant public health concern globally, particularly in the present and post-COVID pandemic era when existing health inequities are amplified [2]. The social determinants of health framework highlights that multiple exposures and contexts shape a child’s health. While different pathways contribute to child health inequities [3], health literacy, reflecting an individual’s capacity to engage with health information and make health decisions [4], plays a crucial role in protecting and maintaining a child’s health in everyday life. Compared with adult health literacy, child health literacy is an emerging field over the last decade. This Special Issue aims to bring together the most recent evidence on child health literacy research and practice to promote child equity.

The Special Issue comprises seven articles, including four original articles, two commentaries, and a systematic review, covering early, middle, and late childhood. Interestingly, there is increasing attention to varieties of children’s health literacy, such as food health literacy, physical health literacy, and oral health literacy. Krenz et al. [5] developed a German Physical Literacy Assessment Tool for children in the context of health promotion and tested it with primary school students in Germany. The work of Kesic et al. [6] investigated gender-specific associations between physical health literacy and generic health literacy among high school students in Croatia. Findings highlight the necessity of independent evaluations of specific and generic health literacy. To provide an overall picture of health literacy research in early childhood, Bánfai-Csonka et al. [7] conducted a systematic review and found that child health literacy mainly focused on specific domains of oral literacy, food literacy, and stroke literacy.

Child health inequities manifest not only in distal outcomes such as body mass index, diet patterns, and physical activity but also in generic health literacy, which is both a determinant of health and an immediate health outcome. Guo et al. [8] investigated health literacy among 650 middle school students in Beijing and found that the distribution of health literacy varied by year level, ethnicity, family composition, family socioeconomic position, and personal health interests. The work of Krenz et al. [5] also showed that children from socioeconomically disadvantaged backgrounds had lower scores of physical health literacy than their advantaged peers.

Health literacy measurement lays the key foundation to investigate child health inequities. The instruments used to measure children’s generic health literacy in this Special Issue include the 15-item Health Literacy Survey (HLS)-Child-Q15 [9], the 47-item HLS-EU-Q47 [6], the 8-item health literacy assessment tool (HLAT-8) [8], the 6-item Newest Vital Sign (NVS) [8], and the 16-item HLS-EU-Q16 [8]. Using different health literacy instruments may help explain observed health inequities across different groups of children and allow for the delivery of interventions that improve health outcomes and address health inequities.

Currently, child health literacy interventions such as school-based programs face implementation challenges (e.g., variability in target groups, timing, and content), which may impact their effectiveness and success. This is one of the interesting objectives of the work of Naccarella and Guo [10], which recommends that researchers and public health practitioners use the health equity implementation framework to better inform the design, implementation, and evaluation of equitable child health literacy interventions, thus contributing to equitable health outcomes.

Finally, in this Special Issue, careful consideration was given to an approach in which children are active participants in health literacy research and practice. The work of Jenkins [11] highlights the need to take an equity-focused approach to establishing a Children’s Advisory Group and drawing on children’s perspectives to understand the meaning of health literacy to them. Investing in health literacy by working with children echoes the child-centered approach, which aims to meet the needs of every child and allows children to take a proactive role in their own health decision making.

This Special Issue highlights that health literacy is closely linked with health equity in children. The studies included in this Special Issue will contribute to and advance our knowledge about specific health literacy, health literacy measurement, and health literacy interventions in children from a health equity perspective. Promoting health literacy in children is essential to achieving an equitable future for all.
